# Complementary feeding hygienic practices and its associated factors among mothers of children aged 6–23 months old in Central Ethiopia, 2023

**DOI:** 10.3389/fpubh.2025.1475315

**Published:** 2025-03-11

**Authors:** Denebo Ersulo Akiso, Sinidu Laelago

**Affiliations:** ^1^Human Nutrition, School of Public Health, College of Medicine and Health Sciences, Wachemo University, Hosanna, Ethiopia; ^2^Disease Prevention and Health Promotion Office, Hosanna, Ethiopia

**Keywords:** complementary feeding, hygienic practice, children, Lemo District, Central Ethiopia

## Abstract

**Background:**

Complementary feeding is a critical period for child development and normal nutritional status, playing a vital role in the prevention of malnutrition and related health issues. However, poor hygiene practices during this phase can lead to foodborne diseases and inadequate nutrient intake.

**Objective:**

To assess poor hygienic practice related to complementary feeding and associated factors among mothers of children aged 6–23 months in Lemo District, 2023.

**Methods:**

A community-based cross-sectional study was conducted from September to November 2023, utilizing a systematic sampling technique. A total of 402 mother–child pairs were randomly selected for participation. Data were collected using a pretested and structured questionnaire and then entered into Epi-Data version 4.6. The data were subsequently exported to SPSS version 26 for analysis. In the multivariable binary logistic regression analysis, variables with a *p*-value less than 0.05 were deemed to be statistically significant.

**Results:**

The prevalence of poor hygienic practices related to complementary feeding among mothers of children aged 6–23 months was found to be 65%. Factors significantly associated with poor hygienic practices of complementary feeding was the absence of a separate kitchen [AOR = 3.17, 95% CI: (1.32, 7.59)], lack of access to a protected source of drinking water [AOR = 8.17, 95% CI: (3.06, 18.7)], and lack of access to media [AOR = 2.12, 95% CI: (1.25, 3.6)].

**Conclusion:**

The study revealed a significant prevalence of inadequate hygienic practices in the context of complementary feeding among mothers. Factors that were identified as significant contributors to the observed poor hygienic practices includes the absence of separate kitchen, unprotected source of drinking water, and a lack of exposure to media.

## 1 Introduction

Complementary Feeding (CF) involves introducing additional foods alongside breast milk. This period is particularly importance due to the inadequate hygiene practices of complementary food observed in many young children with cystic fibrosis, which have been strongly linked to the high incidence of infectious disease (1, 2). Food hygiene is the collection of requirements and safety measures needed to ensure food safety from the point of production to the point of consumption. Food preparation is one of a risk factor for food contamination (3).

Childhood digestive problems are frequently caused by poor hygiene during a child’s CF. (4) Children under the age of two remain vulnerable to gastroenteritis caused by preventable foodborne pathogens because of their developing systems of immunity and vulnerability to sickness with intestinal pathogens. Studies shown that the occurrence of diarrhea rises at the age when complementary foods are frequently introduced, as unhygienic preparation and handling of foods can be a reason for diarrheal pathogens (5). The “first 1,000 days” of life span is the period between the ages of 6 and 23 months, which is the longest. It is crucial to maximize a child’s growth and development during this time to prevent malnutrition and its detrimental effects on adult health. That is why it is referred to as the “window of opportunity” (6).

According to systematic review and meta-analysis study conducted in Ethiopia, pooled prevalence of poor hygienic practice during complementary feeding was 53.47% (7); however, different cross-sectional studies conducted in different regions of Ethiopia showed various prevalence. For examples, 61.1% of Bahir Dar Zuria District (8), 55.1% of Debark town (9), 54.7% of Antsokia Gemza district (10), 66.4% of Tegedie District (11), 60.4% of Rural Kebeles of Harari Region (12) and 57% of Wolaita Sodo Town (13).

Children are particularly susceptible to inadequate hygienic practices associated with the introduction of solid foods. For example, the systematic review and meta-analysis conducted in Ethiopia showed that absences of separate kitchen, rural residence, lack of access to hand washing facility and lack of access to media were significantly associated factors with poor hygienic complementary feeding practices (7).

Undernutrition, which is considered the primary cause of 45% of child deaths under the age of five, is directly linked to the contamination of complementary feeding (14). According to epidemiological research, food may play a greater role in the spread of diarrheal disorders than water. Children under the age of five are thought to bear 40% of the burden of foodborne illnesses in African nations. In Africa, several microbial pathogen infections affect more than 30% of children under the age of five (15).

The World Health Organization (WHO) has outlined guidelines for the preparation of safe food for infants and young children. They advise practicing good hygiene and proper food handling by keeping all food contact surfaces and equipment used in food preparation and serving clean, washing the hands of caregivers and children before food preparation and eating, and storing food safely (16).

However, because of the limited number of studies conducted on the magnitude and associated factors of hygienic practices related to CF among mothers of children aged 6–23 months in Central Ethiopia, the magnitude and factors associated with poor CF hygienic practice have not been adequately addressed. This highlights the importance of providing accurate information regarding individuals in vulnerable age groups. Additionally, no previous studies have been conducted in this specific study area.

## 2 Materials and methods

### 2.1 Study area and period

This study was conducted in Lemo Woreda, which is one of the Woredas in Hadiya Zone, Central Ethiopia. It is geographically located between 70°54′–70°73’ North latitude and 370°89′–38006′ East longitude. The Woreda is bordered by the North and Northwestern parts of Silte Zone and Misha Woreda, the West and Southwest parts of Gombora and Soro Woreda, the Eastern part of Anlemo Woreda, and the Southern and Southwestern parts of Kemebata Zone and Shashogo Woreda. According to the 2007 census, the total population of the Woreda is 160,766, of which 49.36% are male and 50.63% are female. The total number of households is estimated to be 32,809. The total number of mothers who have children aged 6–23 months is estimated to be 7,805 (Lemo Woreda Health Office Report, 2022). The main economic activity in the study area is agriculture (mixed farming). Administratively, the Woreda is divided into 35 Kebeles, and the study was conducted in randomly selected Kebeles. The number of functional health facilities available in the Woreda is one primary hospital, seven health centers, 39 rural health posts, and 11 different private health facilities. The data collection was carried out from September to November 2022.

### 2.2 Study design

A community-based cross-sectional study was conducted.

### 2.3 Source population

All mothers and/ or caregivers who have 6–23 months old children in Lemo Woreda.

### 2.4 Study population

The randomly selected mother–child pairs from 10 kebeles.

### 2.5 Inclusion and exclusion criteria

All mothers/caregivers who have children aged 6–23 months were eligible for this study. However, mothers/caregivers who were ill and unable to respond during the data collection period were excluded from the study.

### 2.6 Sample size determination

The sample size (n) was calculated by considering the following parameters such as proportion (p) = 38.9% from previous study conducted in Northwest Ethiopia (8), Z*α*/2 = 1.96 is the critical point for the standard normal tabulated value at a 95% confidence level, and d = 5% margin of error. $n=\frac{{Z^{2}}_{\alpha_{2}}p\left(1-P\right)}{d^{2}}$ $\frac{{\left(1.96\right)}^{2}0.38.9\left(1-0.38.9\right)}{0.05^{2}}=365.$

The sample size for the first objective with including a 10% non-response rate was 402. For the second objective using epi-info software version 7 (Table 1).

**Table 1:** Sample size calculation for the second objective using epi-info software version 7 of complementary feeding hygienic practice and associated factors among 6–23 months children mothers/caregivers in Lemo Woreda, Hadiya Zone, Central Ethiopia, 2023.

Variables	Power 80%	Confidence level 95%	AOR	Percent in un-exposed	References	Sample size
Residence Rural Urban	80%	95%	7.02	65.4	(17)	80
Private latrine ownership Yes No	80%	95%	4.11	51.3	(16)	90
The presence of a compartment dishwashing facility Yes No	80%	95%	5.70	46.5	(17)	62

When we compare the calculated sample size for both the first objective and second objectives, the sample size for the first objective was larger than the second objective. Therefore, the sample size of the first objective, 402, was taken as the sample size of the population.

### 2.7 Sampling technique and procedure

**Figure 1:**
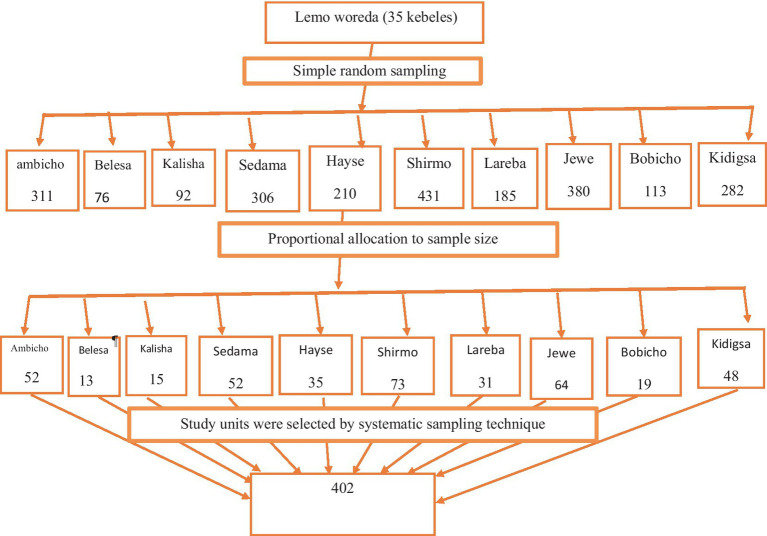
Sampling technique for selection of study participants of complementary feeding hygienic practice and associated factors among 6–23 months children of mothers/caregivers in Lemo Woreda, Hadiya Zone, Central Ethiopia, 2023.

There are 35 kebeles with a total of 8,705 mothers/caregivers who have children aged 6–23 months in Lemo woreda. Out of the 35 kebeles, 10 kebeles were selected using a simple random sampling technique using the lottery method. The sample size was proportionally allocated to each selected kebele based on the size of the population of mothers with 6–23-month-old children in each kebele. The list of mothers with 6–23-month-old children in each kebele was used as a sampling frame, which was found in health posts in the respective kebeles. A systematic random sampling technique was used to select the study participants after proportional allocation. The sampling interval Kth value was determined by dividing the total number of mothers with 6–23 children by the sample size allocated for each kebele. The first participants from 1-kth in each kebele were randomly selected (Figure 1).

### 2.8 Data collection procedure

Data were collected through face-to-face interviews using a structured and pretested questionnaire. The questionnaire was prepared by reviewing previous studies conducted on the hygienic practices of mothers with children aged 6–23 months during complementary feeding. The questionnaire consisted of six parts: socio-demographic, household assets, environment-related variables, maternal knowledge about hygienic complementary feeding, access to media and self-reported hygienic practices of mothers during complementary feeding. Household wealth status was determined based on key household asset ownership variables (such as type of house, livestock, and agricultural land ownership).

### 2.9 Data quality control measures

The questionnaire was prepared in English and translated into Hadiyisa. It was then back-translated into English by another person to ensure consistency and check its conceptual equivalence. Training was provided to the data collectors and supervisor on the aim of the study, inclusion and exclusion criteria, data collection techniques, going through the questionnaires, the art of interviewing, the way of collecting data, and clarification before the actual data collection. Five percent of the sample size was pre-tested outside of the study site in Gibe Woreda to assess its validity and reliability before the actual fieldwork began. The questionnaire was modified based on the results of the pre-test. Daily supervision was conducted by the principal investigator to ensure the completeness and reliability of the data.

### 2.10 Study variables

Dependent variables: Complementary feeding hygienic practices.

Independent variables: Socio-demographic and economic factors, such as age, family size, number of children under two, occupation of the father and mother, level of education of the mother and father, wealth index, and access to media. Behavioural factors, such as mothers’ knowledge, handwashing practice, feeding practice, and home-based water treatment. Environmental factors, such as the presence and type of latrine, the practice of handwashing after using the toilet, source of water, distance to water source, refuse disposal, presence of livestock in the house, presence of a separate kitchen, a three-compartment dishwashing system, and a separate area for storing raw and cooked foods. History of child’s illness, such as diarrhoea, cough, and fever.

### 2.11 Operational definitions

Good complementary feeding hygienic practice: Those study participants who had the correct response to 75% of the questions were reported as having good hygienic practice during complementary feeding; otherwise, they were reported as having poor hygienic practices during complementary feeding.

Wealth index: The composite indicator of socio-economic status, which was computed by the application of principal component analysis.

### 2.12 Data processing and analysis

After completing the data collection, the data were checked for completeness and entered into Epi Data software version 4.6. Then, the data were cleaned and exported to the SPSS statistical package version 26 for further analysis. Descriptive analysis, such as mean, frequency, standard deviation, and percentage was used to examine the overall distribution of the variables under study. Bivariate binary logistic regression was conducted to determine the presence of an association between independent and dependent variables in order to select candidate variables for the multivariable model. Variables with a *p*-value ≤0.25 in the bivariate analysis were included as inputs for the multivariable binary logistic regression analysis. AOR with 95% CI was calculated to indicate the strength of associations. Finally, a p-value of <0.05 in the multivariable binary logistic regression analysis was used to identify variables significantly associated with the dependent variables.

## 3 Results

### 3.1 Socio-demographic characteristics

The study involved 394 mothers with children aged 6–23 months, with a response rate of 98%. The average age of the mothers and caregivers was 30.78 years, with a standard deviation (SD) of 5.01. Among the participants, over half (52.3%) of the mothers or caregivers who took part in the study had no formal education. Likewise, more than half (52.3%) of the households had five or more family members. The majority (85%) of the mothers or caregivers who participated in this study were housewives (Table 2).

**Table 2:** Socio-demographic characteristics of participants in Lemo Woreda, Central Ethiopia, 2023 (394).

Variables	Categories	Frequency	Percentage (%)
Ethnicity	Hadiya	350	88.8
	Kembata	23	5.8
	Others	21	5.4
Religion	Protestant	304	77.2
	Orthodox	35	8.9
	Muslim	46	11.7
	Catholics	9	2.3
Marital status	Married	373	94.7
	Divorced	17	4.3
	Others	4	1
Family size	<5	188	47.7
	≥5	206	52.3
Age of child (month)	6–11	156	39.6
	12–23	238	60.4
Sex	Female	208	52.8
	Male	186	47.2
Educational status of the mother	No formal education	77	19.5
	Primary level	230	58.4
	Secondary level	68	17.3
	Diploma and above	19	4.8
Occupational status of mother	Housewife	335	85
	Civil servant	22	5.6
	Merchant	9	2.3
	Student	28	7.1
Household wealth status	Poor	127	32.2
	Medium	134	34
	Wealthy	133	33.8
Access to media TV/radio	Yes	123	31.2
	No	271	68.8

### 3.2 Prevalence of poor hygienic practice complementary feeding

**Figure 2:**
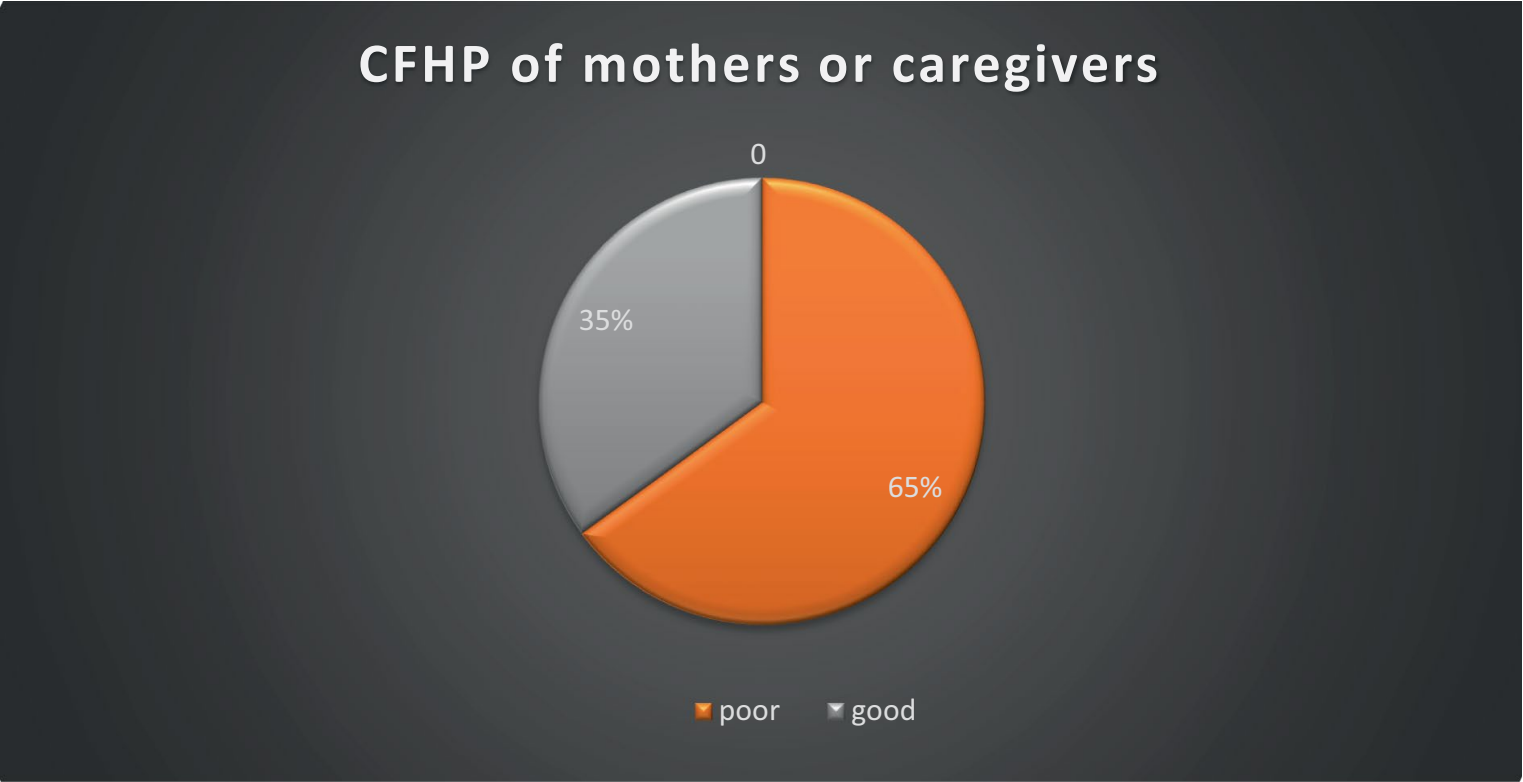
Magnitude of complementary feeding hygienic practice of complementary feeding hygienic practice and associated factors among 6–23 months children in Lemo Woreda, Central Ethiopia, 2023.

Among the 394 mothers/caregivers, 65% revealed poor hygienic practices during the complementary feeding of their children aged 6–23 months Lemo District of Hadiya Zone, Central Ethiopia (Figure 2).

### 3.3 Factors associated with hygienic practices of complementary feeding

In Model-1 six independent predicting factors are included such as access to latrine facility, Functional hand washing facility with soap near the latrine, educational status of mother, availability of separate kitchen, source of water and access to media (Table 3). In Model-2 five independent predicting factors such as functional hand washing facility with soap near the latrine, educational status of mother, availability of separate kitchen, source of water and access to media (Table 4). In Model-3 finally multivariable binary logistic regression analysis, the absence of a separate kitchen for food preparation, unprotected sources of drinking water, and lack of media access were found to be significantly associated with poor complementary feeding hygienic practices (Table 5).

**Table 3:** Model 1: Multiple logistic regression results of complementary feeding hygienic practice among mothers who had 6 to 23 months children in Lemo Woreda, Central Ethiopia, 2023(*n* = 394).

Variables	Categories	Complementary feeding hygienic practice		AOR (95%CI)	p-value
		Good	Poor		
Access to latrine facility	Yes	113	173	1	
	No	25	83	0.28(0.23, 1.04)	0.087
Functional hand washing facility with soap near the latrine	Yes	34	29	1	
	No	91	192	0.46(0.26,0.82)	0.12
Educational status of mother	No formal education	45	34	0.56 (0.45, 3.22)	0.08
	Primary level	112	118	0.97 (0.73, 6.32)	0.31
	Secondary level	42	26	0.34 (0.21, 4.39)	0.54
	Diploma and above	15	4	1	
Drinking water source	Protected	120	121	1	
	Unprotected	18	135	3.35 (1.83,6.63)	0.0001
Separate kitchen for food preparation	Yes	61	150	1	
	No	77	106	3.1 (1.3,7.6)	0.009
Media Access	Yes	59	64	1	
	No	79	192	2.1 (1.25,3.6)	0.005

**Table 4:** Model 2: Multivariable logistic regression results of complementary feeding hygienic practice among mothers who had 6 to 23 months children in Lemo Woreda, Central Ethiopia, 2023(*n* = 394).

Variables	Categories	Complementary feeding hygienic practice		AOR (95%CI)	p-value
		Good	Poor		
Functional hand washing facility with soap near the latrine	Yes	34	29	1	
	No	91	192	0.76(0.54, 1.7)	0.21
Educational status of mother	No formal education	45	34	0.86 (0.75, 4.27)	0.17
	Primary level	112	118	0.73 (0.61, 5.38)	0.07
	Secondary level	42	26	0.51 (0.47, 5.81)	0.24
	Diploma and above	15	4	1	
Drinking water source	Protected	120	121	1	
	Unprotected	18	135	3.35 (1.83,6.63)	0.0001
Separate kitchen for food preparation	Yes	61	150	1	
	No	77	106	3.1 (1.3,7.6)	0.009
Media Access	Yes	59	64	1	
	No	79	192	2.1 (1.25,3.6)	0.005

**Table 5:** Mode1 3: Multivariable logistic regression results of complementary feeding hygienic practice among mothers who had 6 to 23 months children in Lemo Woreda, Central Ethiopia, 2023(*n* = 394).

Variables	Categories	Complementary feeding hygienic practice		AOR (95%CI)	p-value
		Good	Poor		
Drinking water source	Protected	120	121	1	
	Unprotected	18	135	3.35 (1.83,6.63)	0.0001
Separate kitchen for food preparation	Yes	61	150	1	
	No	77	106	3.1 (1.3,7.6)	0.009
Media Access	Yes	59	64	1	
	No	79	192	2.1 (1.25,3.6)	0.005

## 4 Discussion

This study aimed to assess poor hygienic practice of complementary feeding and its factors associated with children aged 6–23 months in Lemo woreda.

The finding of this study showed that the prevalence of poor complementary feeding hygienic practices was found to be 65%. This finding was in line with the study conducted in Bahir Dar Zuria Woreda, Northwest Ethiopia (61.1%) (8), Tegedie Woreda, Northwest Ethiopia (66.4%) (11) and rural Kebeles of the Harari Region, Ethiopia (61%) (12). The explaination may be due to socioeconomic similarity or the living standard of the population.

However, this study prevalence was higher than the prevalence (55%) reported in Debark Town (9). A possible reason for the differences in socioeconomic status is that Debark Town is an urban area, and others living there may have access to better information and infrastructure for complementary feeding. Another study found that the result was higher than the prevalence (49%) reported by a study conducted in the Dedo District, South-western Ethiopia on model and non-model household mothers (17). This discrepancy might be due to differences in the measurement of good hygienic practice and the comparative nature of the previous study, which included model households that have a higher tendency to adopt good food hygiene behaviors.

The odds of poor hygienic practice of complementary feeding were 3 times (AOR = 3.1, 95% CI = 1.32, 7.6) more than among mothers who had no separate kitchen for food preparation compared to mothers who had a separate kitchen. This study consistency with the studies conducted in different region of Ethiopia including Debark Town, Rural Kebeles of Harari, Tegedie District, Ethiopia, systematic review and meta-analysis in Ethiopia, Rural Malawi and Western Kenya (7, 9, 11, 12, 18, 19). The absence of a designated location complicates the proper storage of raw and cooked food, hence heightening the danger of infection. Co-sharing a kitchen for food preparation might result in cross-contamination from raw meats, unclean utensils, or other potentially harmful bacteria, hence increasing the risk of foodborne illness in children.

Mothers/caregivers who used drinking water from unprotected source was 8 times more likely to practice poor hygienic complementary feeding (AOR 8.2; 95% CI = 3.7, 18.7) than mothers who used drinking water from a protected source. This finding was consistent with the studies conducted in the Tegedie District, Wolaita SodoTown, Dedo District and systematic review and meta-analysis, Ethiopia (7, 11, 13, 17). This could be due to that utilizing unprotected water to prepare complementary foods, such as purees or mashed vegetables, can lead to contamination with germs and parasites, hence increasing the child’s risk of infection. This practice may result in recurrent diarrhea, vomiting, malnutrition, and, in extreme instances, death, particularly in youngsters with compromised immune systems.

The odds of practicing poor hygienic complementary feeding among mothers/caregivers who had no media access were 2.1 times (AOR 2.1 95 CI = 1.25, 3.6) higher compared to those mothers who had media access. It was supported by a study conducted in the Wolaita Sodo Town, Antsokia Gemza district, Borecha Woreda, Tegedie District, Dessie Town and Bahir Dar Zuria District, systematic review and meta-analysis Ethiopia (7–10, 13, 20). In the absence of media availability, caregivers may lack exposure to essential information regarding appropriate food handling and sanitary measures vital for complementary feeding, which may result inadequate hygiene habits, such as failing to wash hands before to food preparation, may result in bacterial contamination, potentially leading to diarrhea and other diseases in young children. This could be because the media play a great role in disseminating information related to hygienic practices necessary to implement appropriate complementary feeding.

## 5 Conclusion

From the findings of this study, the prevalence of poor complementary feeding hygienic practices among mothers was a public health concern. The study revealed that a separate kitchen, source of drinking water, and access to media showed a statistically significant association with complementary feeding hygienic practice among mothers who had children aged 6–23 months.

## Data Availability

The raw data supporting the conclusions of this article will be made available by the authors, without undue reservation.

## References

[1] Anand R. Infant and young child feeding In: Henderson P, editor. IAP textbook of pediatrics. 3rd ed. Geneva, Switzerland: WHO (2013). 127–7.

[2] Das S Fahim SM Alam MA Mahfuz M Bessong P Mduma E. Not water, sanitation and hygiene practice, but timing of stunting is associated with recovery from stunting at 24 months: results from a multi-country birth cohort study. Public Health Nutr. (2021) 24:1428–37. doi: 10.1017/S136898002000004X, PMID: 32404220 PMC8025093

[3] Haldar RN. World health day 2015-food safety. Indian journal of physical medicine and rehabilitation. (2015) 26:1.

[4] Ehiri JE Azubuike MC Ubbaonu CN Anyanwu EC Ibe KM Ogbonna MO. Critical control points of complementary food preparation and handling in eastern Nigeria. Bull World Health Organ. (2001) 79:423–33. PMID: 11417038 PMC2566429

[5] Mattioli MC Pickering AJ Gilsdorf RJ Davis J Boehm AB. Hands and water as vectors of diarrheal pathogens in Bagamoyo, Tanzania. Environmental Science & Technology. (2013) 47:355–63. doi: 10.1021/es303878d, PMID: 23181394

[6] Bizzego A Gabrieli G Bornstein MH Deater-Deckard K Lansford JE Bradley RH. Predictors of contemporary under-5 child mortality in low-and middle-income countries: a machine learning approach. Int J Environ Res Public Health. (2021) 18:1–12. doi: 10.3390/ijerph18031315 PMC7908191 33535688

[7] Zeleke AM Tassew WC Ayale Ferede Y Andargie TM. Hygienic practices and factors of complementary food preparation among mothers of children aged 6–24 months in Ethiopia: a systematic review and meta-analysis. Front Food Sci Technol. (2024) 4:1240979. doi: 10.3389/frfst.2024.1240979

[8] Demmelash AA Melese BD Admasu FT Bayih ET Yitbarek GY. Hygienic practice during complementary feeding and associated factors among mothers of children aged 6-24 months in Bahir Dar Zuria District, Northwest Ethiopia, 2019. J Environ Public Health. (2020) 2020:1–7. doi: 10.1155/2020/2075351

[9] Zeleke AM Bayeh GM Azene ZN. Hygienic practice during complementary food preparation and associated factors among mothers of children aged 6 – 24 months in debark town, Northwest Ethiopia, 2021: an overlooked opportunity in the nutrition and health sectors. PLoS One. (2022) 17:1–17. doi: 10.1371/journal.pone.0275730 PMC9733846 36490237

[10] Tadegew G Chane T Bogale EK. Hygienic complementary feeding practice and its associated factors among mothers having children aged 6–23 months in Antsokia Gemza district, Ethiopia: a cross-sectional survey. BMJ Public Health. (2024) 2:307. doi: 10.1136/bmjph-2023-000307 PMC11816969 40018249

[11] Yallew WW Azanaw J. (2021). Complementary feeding hygiene practice and associated factors among mothers with children aged 6–24 months in tegedie district, Northwest Ethiopia: Community-based cross-sectional study. pp.1–22.

[12] Fufa DD Abhram A Teshome A Teji K Abera F. Hygienic practice of complementary food preparation and associated factors among mothers with children aged from 6 to 24 months in rural Kebeles of Harari. Food Sci Technol. (2020) 8:34–42. doi: 10.13189/fst.2020.080203

[13] Kassie GA Gebeyehu NA Gesese MM Chekol Abebe E Mengstie MA Seid MA. Hygienic practice during complementary feeding and its associated factors among mothers/caregivers of children aged 6–24 months in Wolaita Sodo town, southern Ethiopia. SAGE Open Med. (2023) 11:20503121231195416. doi: 10.1177/20503121231195416, PMID: 37655302 PMC10467249

[14] Watson S Gong YY Routledge M. Interventions targeting child undernutrition in developing countries may be undermined by dietary exposure to aflatoxin. Crit Rev Food Sci Nutr. (2017) 57:1963–75. doi: 10.1080/10408398.2015.1040869, PMID: 26176888

[15] Oladayo T Miteu G Addeh I Folayan E Olayinka T Adegboyega J. Most prominent factors of food poisoning in africa: Nigeria based perspective. IPS J Nutr Food Sci. (2022) 1:11–7. doi: 10.54117/ijnfs.v1i1.1

[16] World Health Organization. WHO guideline for complementary feeding of infants and young children 6–23 months of age. Geneva: World Health Organization (2023).

[17] Birhanu MT Liga AD Jabir YN. Practices of hygiene during complementary food feeding and associated factors among women with children aged 6−24 months in Dedo district, Southwest Ethiopia: a cross-sectional study. Health Sci Rep. (2023) 6:e1771. doi: 10.1002/hsr2.1771, PMID: 38111740 PMC10725999

[18] Chidziwisano K Slekiene J Kumwenda S Mosler HJ Morse T. Toward complementary food hygiene practices among child caregivers in rural Malawi. Am J Trop Med Hyg. (2019) 101:294–303. doi: 10.4269/ajtmh.18-0639, PMID: 31237230 PMC6685574

[19] Ogutu EA Ellis A Rodriguez KC Caruso BA McClintic EE Ventura SG. Determinants of food preparation and hygiene practices among caregivers of children under two in Western Kenya: a formative research study. BMC Public Health. (2022) 22:1865. doi: 10.1186/s12889-022-14259-6, PMID: 36203140 PMC9535979

[20] Addis AT Dawed YA Yimer GM Adem YF. Complementary food hygiene practice and associated factors among mothers with children aged 6–23 months in Dessie Zuria, south Wollo zone, Amhara, Ethiopia, 2023. Front Nutr. (2024) 11:1465008. doi: 10.3389/fnut.2024.1465008, PMID: 39555192 PMC11565939

